# Legacy 4(1*H*)‑Quinolone Scaffolds Activity against Acute and Chronic *Toxoplasma gondii* Infection

**DOI:** 10.1021/acsinfecdis.5c01074

**Published:** 2026-05-26

**Authors:** Melissa A. Sleda, Khaly Diagne, Victoria Mills Clifton, Baihetiya Baierna, Roman Manetsch, Silvia N. J. Moreno

**Affiliations:** † Center for Tropical and Emerging Global Diseases, 1355 University of Georgia, Athens, Georgia 30602, United States; ‡ Department of Chemistry and Chemical Biology, 50919 Northeastern University, Boston, Massachusetts 02115, United States; § Department of Cellular Biology, University of Georgia, Athens, Georgia 30602, United States; ∥ Department of Pharmaceutical Sciences, Northeastern University, Boston, Massachusetts 02115, United States; ⊥ Center for Drug Discovery, Northeastern University, Boston, Massachusetts 02115, United States; # Barnett Institute of Chemical and Biological Analysis, Northeastern University, Boston, Massachusetts 02115, United States

**Keywords:** Toxoplasma gondii, chronic infection, tissue cysts, bradyzoites, quinolones, electron transport chain

## Abstract

*Toxoplasma gondii* is a protozoan parasite capable of infecting most warm-blooded animals, including humans, and can cause severe disease in immunocompromised individuals and the developing fetus. Current treatments for toxoplasmosis are effective only against the acute stage of infection and have limited or no activity against the latent bradyzoite stage found within tissue cysts. The mitochondrion of *T. gondii* is a validated drug target, and the clinically used drug atovaquone acts by inhibiting the mitochondrial electron transport chain (ETC) at the coenzyme Q:cytochrome *c* oxidoreductase (*bc*_1_ complex). In this study, we evaluate two legacy 4­(1*H*)-quinolones, ICI 56,780 and WR 243246, previously shown to inhibit the *Plasmodium falciparum* *bc*_1_ complex, for their efficacy against *T. gondii*. Both compounds inhibit tachyzoite growth with low-nanomolar EC_50_ values (0.34 nM for ICI 56,780 and 24 nM for WR 243246) and disrupt parasite mitochondrial function by blocking cytochrome *c* reduction and collapsing the mitochondrial membrane potential. Importantly, ICI 56,780 protects mice from lethal infection with type I RH tachyzoites. It also exhibits potent activity against chronic-stage parasites, reducing cyst size and bradyzoite viability in vitro and showing low-nanomolar EC_50_ values against in vivo-derived bradyzoites (EC_50_: 3.9 nM). In mice chronically infected with *T. gondii*, treatment with ICI 56,780 significantly decreases brain cyst burden. Although these 4­(1*H*)-quinolones display some pharmacokinetic limitations, our findings highlight their potential as promising chemotypes active against both acute and chronic stages of *T. gondii* and provide a basis for future medicinal chemistry efforts to improve drug-like properties while preserving or enhancing antibradyzoite activity.

## Graphical abstract

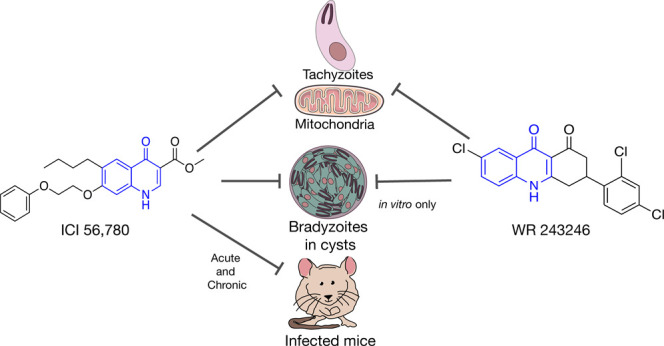

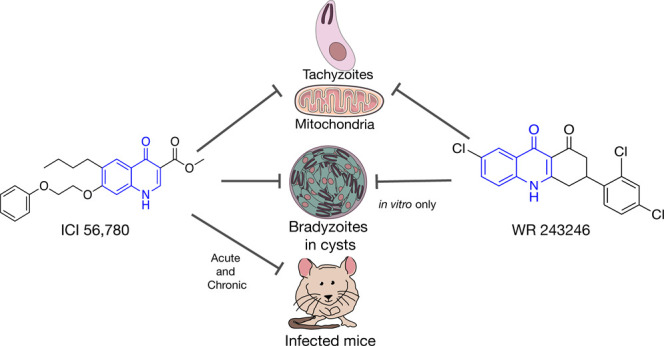

## Introduction

*Toxoplasma gondii* is an obligate intracellular parasite that infects approximately one-third of the global human population.^1^ Upon infection, *T. gondii* replicates as tachyzoites, the fast-replicating stage that destroys host tissues and disseminates throughout the body. In immunocompetent individuals, the immune response controls the acute infection, and tachyzoites differentiate into bradyzoites, a slow-growing form that resides within tissue cysts. These cysts can persist in the host for life, mainly in the brain and muscle tissues.^2^ In immunosuppressed individuals, loss of immune control allows tachyzoites arising from bradyzoites to expand and cause severe disease. Current treatments for toxoplasmosis, including pyrimethamine and sulfadiazine, are effective against tachyzoites^3^ but have minimal activity against the bradyzoite stage within tissue cysts.^3,5^ The last clinically approved drug, atovaquone (ATQ), targets the mitochondrial *bc*_*1*_ complex^6,7^ and shows some effect against bradyzoites, but fails to completely eliminate tissue cysts during chronic infection.^8^

Mitochondrial metabolism has emerged as an important vulnerability of *T. gondii* for both acute and chronic stages of infection. Although bradyzoites were long thought to exhibit reduced metabolic activity compared with tachyzoites, raising questions about the extent of mitochondrial metabolism in this stage,^9,10^ several studies have shown that mitochondrial pathways remain functionally relevant during the chronic stage.^11^ Consistent with this, the cytochrome *bc*_1_ inhibitor ATQ reduces tissue cyst numbers and mitigates neuropathology in chronically infected mice.^8,12^ More recent research further validates the mitochondrion as a potential target during bradyzoite development, although direct evidence of mitochondrial inhibition in this stage remains limited.^13,14^

Our laboratory identified a lipophilic bisphosphonate that inhibits ubiquinone (UQ) synthesis, an essential component of the ETC, and demonstrated that it is highly effective at protecting mice against a lethal infection with *T. gondii*,^15^ and significantly reduces tissue cyst burden.^16^ Other inhibitors of the *bc*_1_ complex, like endochin-like quinolones (ELQs), similarly decrease tissue cyst burden in chronically infected mice.^4,17,18^ Together, these findings support the sensitivity of bradyzoites to compounds targeting mitochondrial function and suggest the ETC as a promising target for therapeutic intervention.

Quinolone compounds have been shown to inhibit DNA replication by targeting DNA gyrase and topoisomerase IV.^19^ In addition, quinolone derivatives have also been reported to induce apoptosis in cancer cells,^20,21^ modulate bacterial redox homeostasis,^22^ and chelate metal ions, which can alter enzymatic functions and metabolic processes.^23^ The 4­(1*H*)-quinolone scaffold has re-emerged as a promising chemotype for antiparasitic drug development, with historical roots in the antimalarial compound ICI 56,780 (Figure 1A). Initially discovered in the mid-20th century, ICI 56,780 demonstrated potent activity against *Plasmodium* spp. but was ultimately discontinued due to poor aqueous solubility and rapid emergence of resistance in vivo.^24^ Despite these limitations, its ability to inhibit the parasite mitochondrial cytochrome *bc*_1_ complex provided a valuable pharmacological foundation. Extensive structure–activity (SAR) and structure–property (SPR) relationship studies by Manetsch and colleagues refined this scaffold, yielding analogs with nanomolar potency against multidrug-resistant *Plasmodium falciparum*, in vivo efficacy across parasite life stages, and *bc*_1_ complex inhibition as the confirmed mechanism of action.^24–30^ To improve metabolic and physicochemical properties, 7-(2-phenoxyethoxy)- and 7-piperazine-substituted 4­(1*H*)-quinolones were synthesized and shown to possess nanomolar potency against multidrug-resistant *P. falciparum*, alongside robust in vivo efficacy.^24,30^ These optimized analogs retained activity against both blood- and liver-stage parasites and displayed oral bioavailability and curative potential in murine malaria models. Mechanistic studies confirmed that these compounds act via inhibition of the cytochrome *bc*_1_ complex, consistent with their structural lineage. Given the conserved mitochondrial ETC machinery across apicomplexan parasites, this class of compounds warrants further investigation against related pathogens such as *T. gondii*.

**Figure 1:**
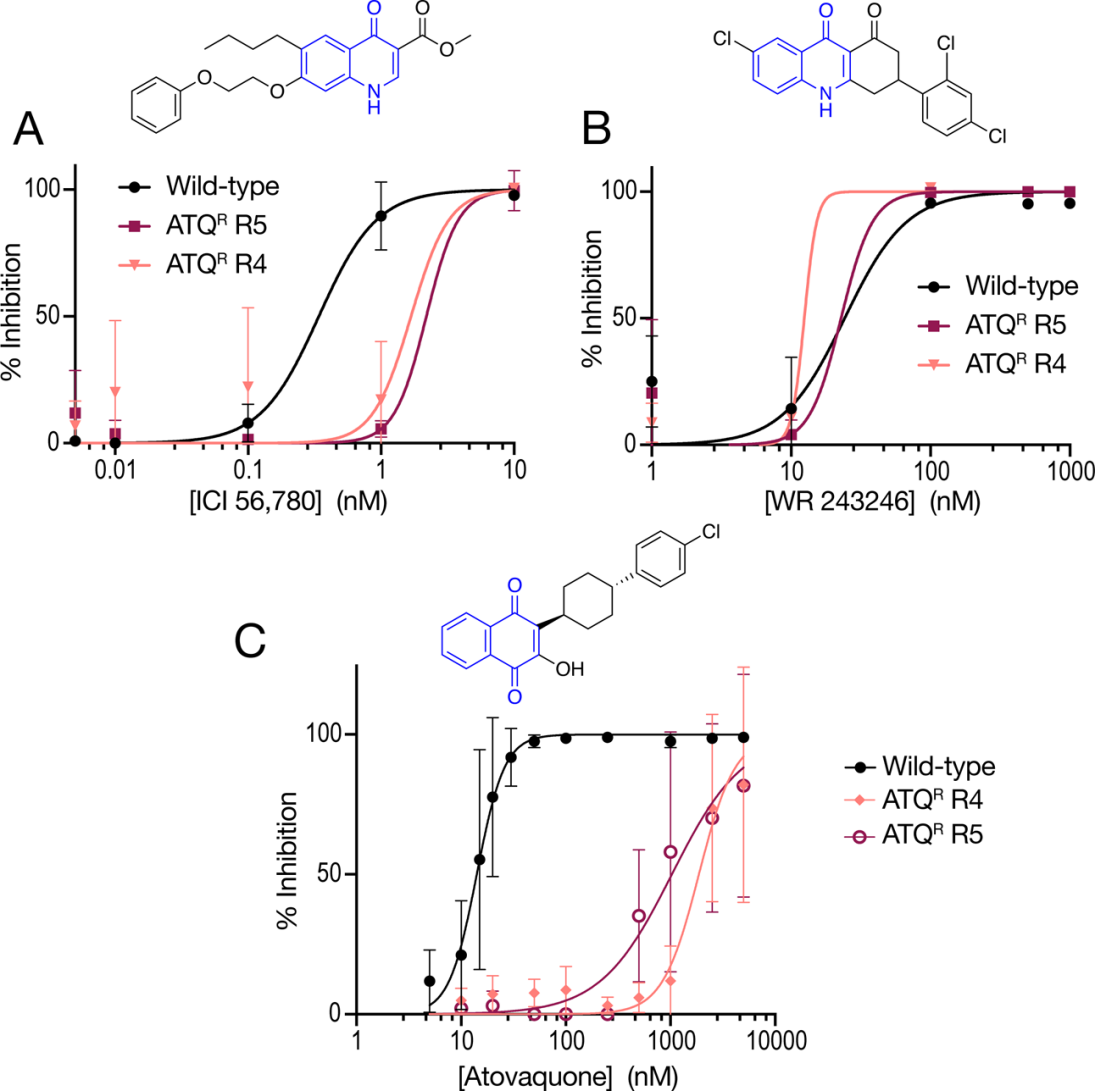
Inhibition of tachyzoite growth by ICI 56,780 and WR 243246. (A). EC_50_ curves for ICI 56,780 in RH-RFP (type I) wild-type tachyzoites and ME49 (type II) ATQ^R^ strains R4 and R5. Growth was measured by quantifying red fluorescence of RH-RFP or crystal violet absorbance in the R4 and R5 strains. (B). EC_50_ curves for WR 243246 in RH-RFP and ATQ^R^ strains R4 and R5. (C). EC_50_ curves for ATQ in RH-RFP and ATQ^R^ strains R4 and R5. Each curve represents the average from 3 biological replicates. A nonlinear regression was used to calculate EC_50_ using Prism.

The dihydroacridinedione scaffold has also contributed to antimalarial drug discovery, with WR 243246 serving as an early lead compound. Further optimization of 1,2,3,4-tetrahydroacridin-9­(10*H*)-ones (THAs) produced derivatives with enhanced potency and improved pharmacokinetic profiles, including efficacy against chloroquine-resistant *P. falciparum* strains.^28^ These findings support the exploration of THA analogs against other apicomplexan pathogens.

The mitochondrion of *T. gondii* has emerged as a promising target for therapeutic intervention, given its essential role in parasite survival and its divergence from mammalian host mitochondria.^31,31b^ Several studies have demonstrated that inhibitors targeting the mitochondrial ETC, such as ATQ and ELQs, reduce tissue cyst burden in murine models, underscoring the ETC’s critical function in bradyzoite viability.^18^ Additional mitochondrial enzymes with no mammalian homologues, such as malate/quinone oxidoreductase (MQO),^32^ provide opportunities for selective targeting. The identification of mitochondrial proteins such as TgPRELID, which has been linked to resistance to mitochondrial inhibitors, further highlights the importance of mitochondrial pathways in parasite physiology and drug response.^33^ Collectively, these findings support continued exploration of mitochondrial pathways as promising targets for developing effective treatments against chronic toxoplasmosis.

In this study, we explore the efficacy of two 4­(1*H*)-quinolones, ICI 56,780 and WR 243246, against *T. gondii*. Both ICI 56,780 and WR 243246 differ from the ELQ quinolone series in the position of the main substituent in the quinolone core. We investigate their impact on both tachyzoite and bradyzoite stages, assess their mechanism of action, and evaluate their effectiveness against ATQ-resistant strains. These findings contribute to the development of more effective therapies targeting chronic *T. gondii* infection.

## Results

### Inhibition of *T. gondii* Tachyzoite Proliferation and Mitochondrial Function

We tested the activity of the legacy 4­(1*H*)-quinolones ICI 56,780 and WR 243246 against the in vitro proliferation of *T. gondii* tachyzoites of the type I RH strain and two Type II ATQ-resistant (ATQ^R^) strains (R4 and R5).^7^ We found that ICI 56,780 inhibits *T. gondii* RH with an EC_50_ of 0.34 nM, and it retained efficacy against both ATQ^R^ strains with EC_50_ values of 1.68 nM and 2.19 nM against R4 and R5, respectively (Figure 1A and Table 1). WR 243246 is also effective against RH with an EC_50_ of 24.2 nM and is equally effective against R4 and R5 with EC_50_ values of 22.7 nM and 12.5 nM, respectively (Figure 1B and Table 1). For comparison, ATQ displayed an EC_50_ of 14.1 nM against wild-type RH, but resistance in the R4 and R5 strains resulted in markedly increased EC_50_ values of 1.87 μM and 1.03 μM, respectively (Figure 1C).

**Table 1:** EC_50_s Values against Tachyzoites and Bradyzoites, Cytotoxicity in Host Cells (Fibroblasts and Glial Cells), and Physiochemical Properties of ICI 56,780 and WR 243246

	units/measure	
assay/property	ICI 56,780	WR 243246
EC_50_ (nM) against RH tachyzoites	0.34 ± 0.24	24.2 ± 19.1
EC_50_ (nM) against ME49-RFP tachyzoite growth	0.32 ± 0.14	17.2 ± 19.6
EC_50_ (nM) against ME49 R4 ATQ^R^ tachyzoites	1.68 ± 0.84	22.7 ± 39.1
EC_50_ (nM) against ME49 R5 ATQ^R^ tachyzoites	2.19 ± 1.11	12.5 ± 0.04
EC_50_ (nM) against RFP- ME49-Bradyzoite viability	3.92 ± 3.38	1271 ± 734.5
cytotoxicity (μM) in HFF	>50	>50
cytotoxicity (μM) in BV-2	>50	82.6
selectivity index (SI) wild-type tachyzoites in BV-2	>147,058	3413
aqueous solubility (μM)	<1.25	ND
human liver microsomes, clint (μL/min/mg)	<3.95	6.2
human liver microsomes *T*-half (min)	>395	232.2
rat hepatocytes, clint (μL/min/10^6^ cells)	7.95	<1.2
rat hepatocytes, *T*-half (min)	87.2	>592
log *D*	4.85	3.46

To investigate the effects of ICI 56,780 and WR 243246 on mitochondrial function, we performed a succinate-cytochrome c reductase assay, which measures electron flow from Complex II through UQ to Complex III using succinate as the substrate (Figure 2A). Both compounds inhibited cytochrome *c* reduction similarly to ATQ, consistent with inhibition of the ETC (Figure 2B).

**Figure 2:**
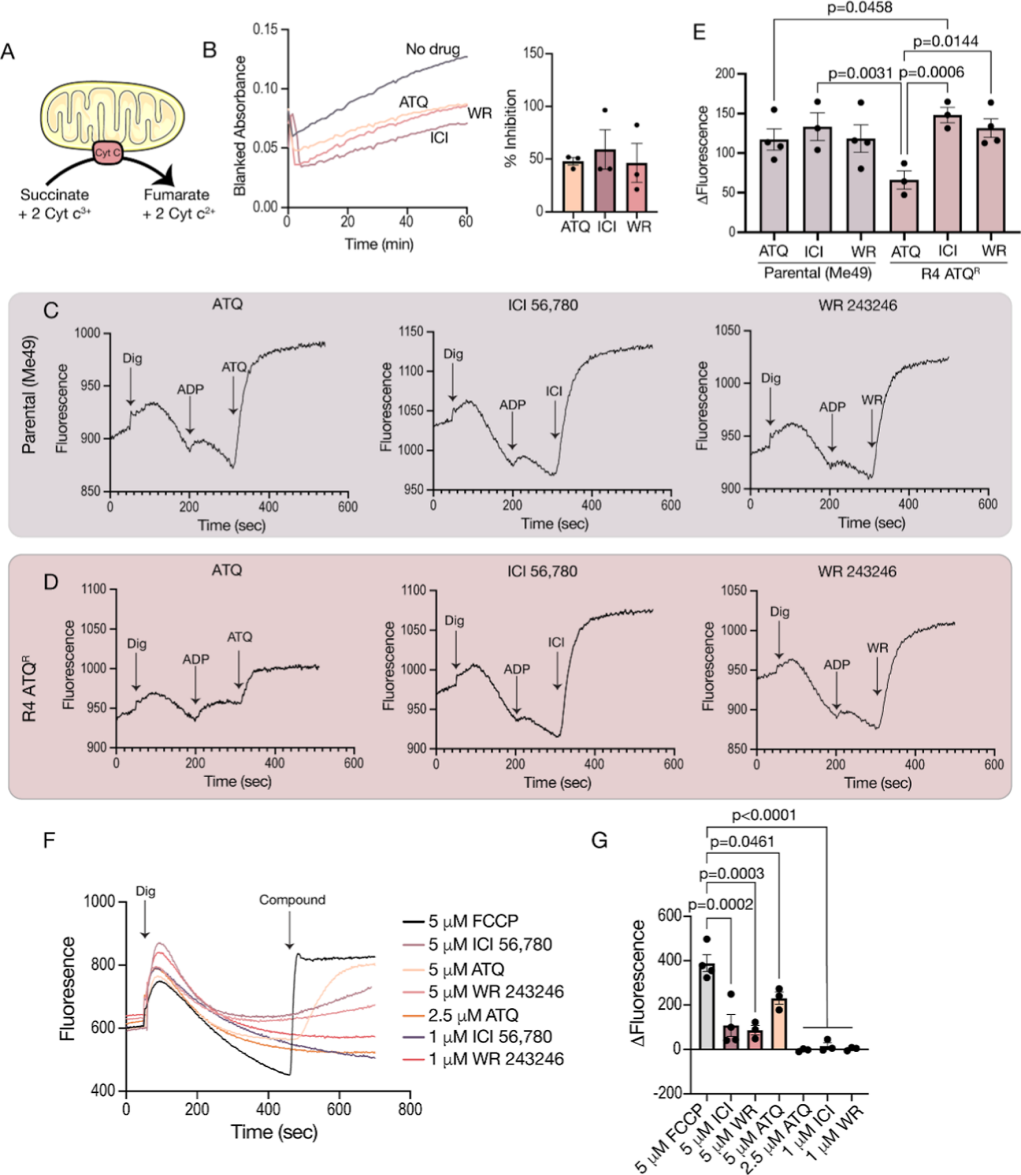
Effects of ICI 56,780 and WR 243246 on mitochondrial electron transport and membrane potential. (A). Schematic of the cytochrome *c* reduction assay. (B). Representative traces for the cytochrome *c* reduction activity of *T. gondii* mitochondrial fractions in the absence of additions or in the presence of the indicated inhibitors at 100 nM. The bar graph shows percent inhibition values representing the averages of three independent biological replicates. (C). Representative traces of mitochondrial membrane potential measured with safranin O (2.5 μM) in the parental cell line (ME49) during real-time addition of the indicated compounds (Dig: Digitonin, 10 μM; ADP, 10 μM). (D). Representative traces of mitochondrial membrane potential in the R4 ATQ^R^ cell line during real-time addition of each drug. (E). Quantification of the average change in fluorescence after each addition (2.5 μM ATQ; 1 μM ICI 56,780 and WR 243246), from at least three independent biological replicates. (F). Representative tracings of hTert fibroblast mitochondrial membrane potential measurements. Digitonin (50 μM) was added at 50 s, and compounds (at indicated concentrations) were added at 450 s. FCCP at 5 μM was used as a control for maximum depolarization. (G). Quantification of the average change in fluorescence after drug addition, from 3 independent biological replicates. Statistical analysis in E and G was performed using two-way ANOVA.

Additionally, we analyzed the mitochondrial membrane potential (ΔΨ_m_) using safranin O in digitonin-permeabilized tachyzoites in the presence of succinate as substrate.^34^ Following digitonin permeabilization, Safranin O accumulates within polarized mitochondria, leading to a characteristic decrease in fluorescence that reflects the membrane potential. Upon addition of ADP, the ATP synthase is activated, which utilizes the proton gradient and induces partial mitochondrial depolarization. This depolarization leads to the redistribution of safranin O, resulting in a corresponding change in fluorescence. To evaluate the effect of the test compounds on mitochondrial membrane potential, we added them at 1 μM and monitored the resulting changes in safranin O fluorescence. Both ICI 56,780 and WR 243246 depolarized the mitochondrial membrane potential similarly to ATQ used as a control (Figure 2C–E). Fluorescence responses to ATQ were markedly reduced in the ATQ^R^ cell line.

However, both ICI 56,780 and WR 243246 showed similar activity in the ATQ^R^ strain compared to the parental ME49 strain (Figure 2E). This suggests that, although these compounds inhibit the ETC like ATQ, they likely target a different site within the cytochrome *bc*_1_ complex.

Additionally, we tested the effects of these compounds on the membrane potential of mammalian host cells (hTERT fibroblasts) at the same concentrations that inhibit parasite mitochondrial membrane potential. No significant change in membrane potential was observed at concentrations similar to those that inhibit parasite mitochondrial membrane potential (Figure 2F,G). However, when the concentration of each compound was increased to 5 μM, a significant reduction in the host mitochondrial membrane potential was detected (Figure 2F,G). FCCP was used as a depolarizing control and defined as 100% inhibition of the membrane potential.^35^ These results indicate that the compounds are unlikely to be toxic to host mitochondria at concentrations that disrupt parasite mitochondrial membrane potential.

### Physicochemical Properties and Compound Stability

Considering that we aimed to evaluate these compounds in both mouse models of toxoplasmosis, acute and chronic, we next profiled key physicochemical and metabolic properties of ICI 56,780 and WR 243246 to identify potential liabilities that might limit efficacy studies in animals. Both compounds are lipophilic and poorly soluble in aqueous media, with logD values of 4.85 for ICI 56,780 and 3.5 for WR 243246. The aqueous solubility of ICI 56,780 was <2 μM, while the solubility of WR 243246 was too low to be reliably determined in the solubility assay used. Both compounds are also predicted to exhibit high plasma protein binding, which may limit free drug exposure in vivo, reducing their suitability for detailed pharmacokinetic profiling in mice at this stage. Consequently, future structure–activity relationship optimization will focus on improving the physicochemical properties of these chemotypes to enable oral delivery and to achieve plasma concentrations sufficient to produce robust in vivo efficacy. Metabolic stability was determined using rat hepatocytes and human liver microsomes, with rat serving as a rodent comparator commonly used in preclinical studies and human microsomes providing translational insight into potential human metabolic stability (Table 1). ICI 56,780 exhibited good metabolic stability in both human microsomes and in rat hepatocytes with a very low intrinsic clearance (Clint values ≤8 μL/min/10^6^ cells and half-life values ≥90 min). WR 243246, was similarly stable in human microsomes (Clint = 6.2 μL/min/mg, *t*_1/2_ > 592 min) but was significantly less stable in rat hepatocytes, with a very short half-life of less than 2 min, Table 1 indicating species-specific metabolic instability. Importantly, these profiling studies indicate that while the basic physicochemical properties of both ICI 56,780 and WR 243246 are not optimal, they are sufficient to support in vivo efficacy studies.

We also evaluated host cytotoxicity in HFF fibroblasts and BV-2 microglial cells. No evidence of toxicity was observed for either compound in HFF fibroblasts (Tables 1 and S1). In BV-2 microglia, however, cytotoxicity was observed for ATQ (CC_50_ = 33.24 μM) and WR 243246 (CC_50_ = 82.6 μM) (Tables 1 and S2). No evidence of toxicity was detected for ICI 56,780, preventing determination of a CC_50_ (Table S2).

### Activity of ICI 56,780 and WR 243246 in the Acute Infection Model

We next evaluated in vivo efficacy using an acute infection model in Swiss Webster mice. Female Swiss Webster mice were infected intraperitoneally (i.p.) with 100 RH-RFP *T. gondii* tachyzoites, and treatment with the test compounds was initiated 6 h postinfection at the indicated doses. Treatment continued for 10 consecutive days, during which body weight and survival were monitored (Figure 3A). ICI 56,780 protected mice from lethal infection at doses as low as 1 mg/kg/day and provided complete protection at 10 mg/kg/day (Figure 3B). For comparison, atovaquone (ATQ) has been reported to provide full protection in this model at 75 mg/kg, while at 4.6 mg/kg it fails to protect, resulting in 100% mortality.^36^ Weight monitoring revealed that treated mice experienced transient weight loss around days 8–12, coinciding with visible signs of illness. However, the mice recovered and regained weight over the course of the experiment (Figure S1A–C). To verify that the surviving mice had been infected and had mounted an immune response, we collected serum 28 days postinfection (dpi) and tested for *T. gondii*-specific antibodies, before challenging the animals with 10,000 RH-RFP tachyzoites at 30 dpi (Figure S1D–F). Body weight and survival were monitored for 3 weeks before the animals were euthanized. We also tested WR 243246 in the acute lethal infection model. However, only a single mouse treated with 4 mg/kg/day survived the infection, while all the other treated animals succumbed to infection (Figures 3C and S1G–I). Increasing the doses to 5, 10, and 20 mg/kg/day did not improve the survival outcome (Figures 3D and S1H). This lack of efficacy at higher doses may reflect limited bioavailability of WR 243246, an issue that we plan to address through the development of improved analogs.

**Figure 3:**
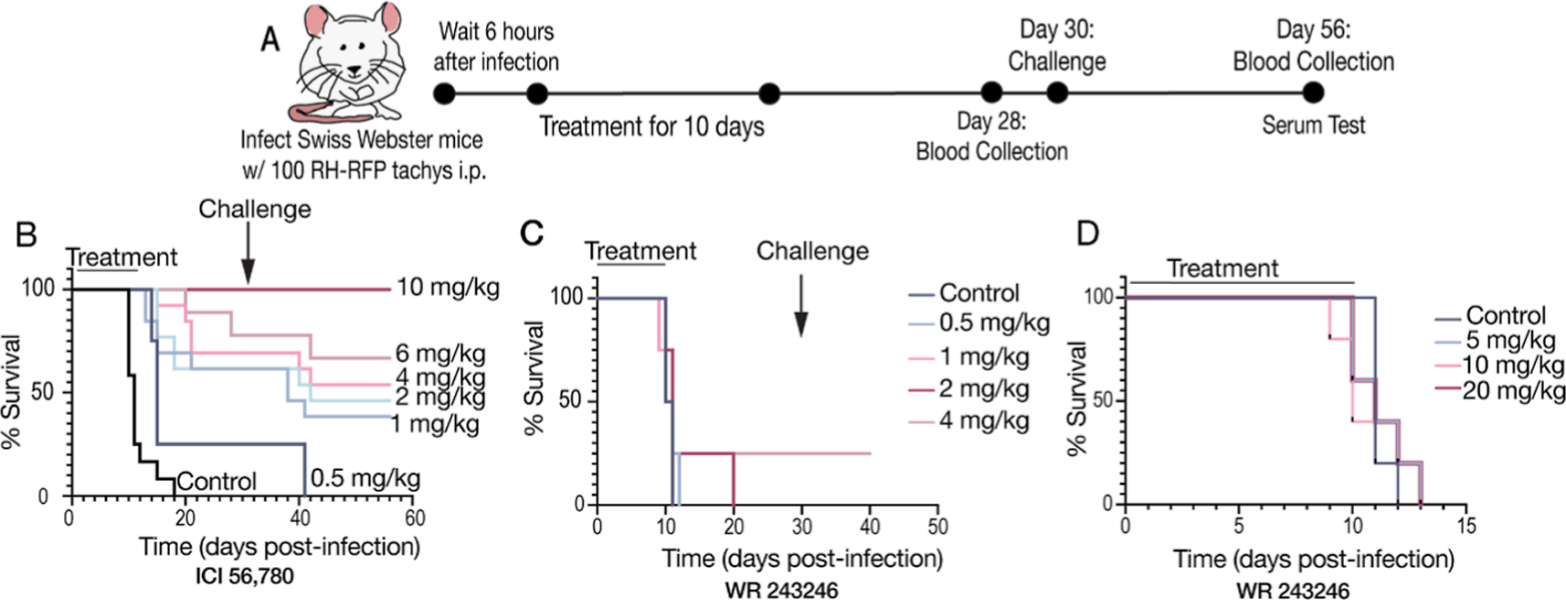
Acute *T. gondii* infection model and treatment in mice. (A). Schematic of the acute infection model. Swiss Webster mice were infected with 100 RH-RFP tachyzoites i.p.. Treatment was initiated 6 h postinfection and administered daily by i.p. injection at the doses indicated. At 28 dpi, blood was collected for serum testing, and mice were challenged with 10,000 RH-RFP tachyzoites at day 30. Three weeks after the challenge, mice were euthanized, and the final blood was collected for serum testing. (B). Survival curve for 3 biological replicates of mice treated with ICI 56,780 with 0.5, 1, 2, 4, 6, and 10 mg/kg/day. Kaplan–Meier survival analysis showed significant differences among groups (log-rank test, p=0.0001). (C). Survival curve of one biological replicate of mice treated with WR 243246 at 0.5, 1, 2, and 4 mg/kg. (D). Survival curve for one biological replicate of mice treated with WR 243246 at 5, 10, and 20 mg/kg.

### Activity of ICI 56,780 and WR 243246 against In Vitro-Derived Bradyzoites

To determine whether the compounds also affect the chronic stage, we evaluated their activity against bradyzoites. We first tested them against in vitro-derived bradyzoites using a previously established protocol to differentiate ME49 using compound 1 (4-[2-(4-fluorophenyl)-5-(1-methylpiperidine-4-yl)-1H-pyrrol-3-yl]­pyridine) and low CO_2_ growth conditions.^37^ After differentiation of tachyzoites into bradyzoites, the cultures were treated with 30 nM of each compound for 4 days. We used fluorescence lectin staining with rhodamine-conjugated *Dolichos biflorus* agglutinin (DBA) to visualize tissue cysts, as DBA labels the cyst wall,^38^ together with an immunofluorescence assay using anti-BAG1 (bradyzoite antigen 1) antibodies. Additionally, we stained the cysts with anti-SAG1 (tachyzoite surface antigen 1), since SAG1 expression is associated with immature or incompletely differentiated cysts (Figure 4A, B). We found that both ICI 56,780 and WR 243246 reduced cyst size (Figure 4B). Notably, WR 243246-treated cultures showed internal spaces within the cysts not occupied by parasites, indicating disruption of cyst organization (Figure 4B). We next tested bradyzoite viability by mechanically disrupting the in vitro-derived cysts, followed by pepsin treatment to release bradyzoites and eliminate any remaining tachyzoites. The recovered bradyzoites were then used in plaque assays to evaluate their viability (Figure 4C). Under these conditions, surviving bradyzoites convert to tachyzoites and initiate lytic cycles that disrupt the host monolayer, forming characteristic plaques. The number of plaques formed reflects the number of initial viable bradyzoites present in the sample.

**Figure 4:**
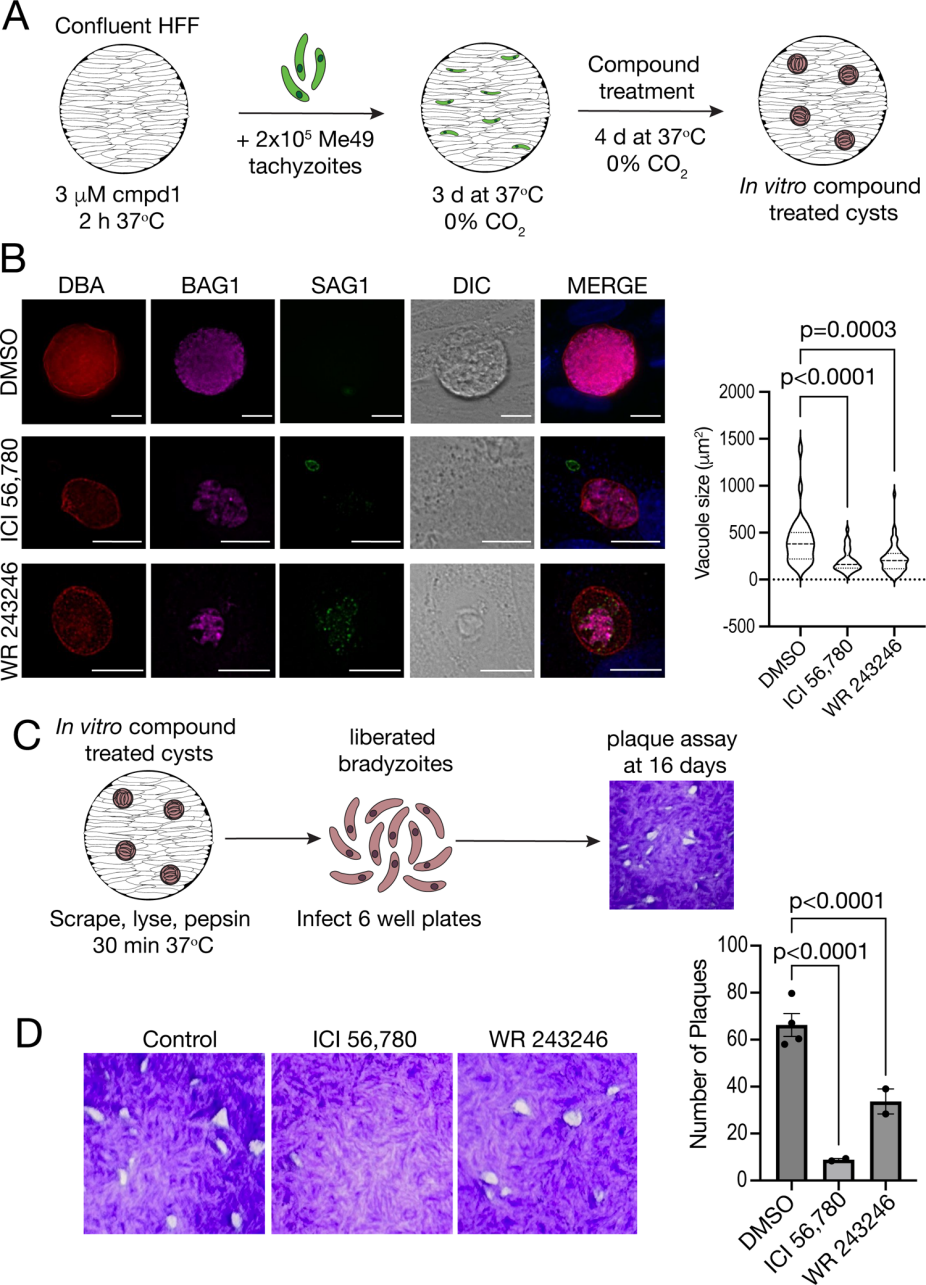
Activity of ICI 56,780 and WR 243246 against in vitro-derived bradyzoites. (A). Schematic showing the protocol for in vitro differentiation of ME49 tachyzoites into bradyzoites. HFF monolayers were incubated with compound 1, 2 h before infection with 2 × 10^5^ ME49 tachyzoites. The cells were incubated for 3 days at ambient CO_2_. Compounds were then added at 30 nM and incubated for an additional 4 days at ambient CO_2_ before fixation and staining with markers for the cyst wall (DBA), bradyzoites (BAG1), and tachyzoites (SAG1). (B). Representative images for each condition, scale bar is 10 μm. The size of the cysts was measured using ImageJ and averaged from 3 biological replicates. (C). Schematic showing the protocol used for the in vitro bradyzoite viability assay. First, tachyzoites were differentiated as described in A, then the compound-treated cysts were syringe lysed and treated with pepsin to release bradyzoites. The bradyzoites are then plated onto confluent HFF monolayers at 10,000 bradyzoites per well and allowed to grow under normal growth conditions with compound-free DMEM for 16 days. (D). Representative images of plaques for each condition. The bar graph shows the average number of plaques formed from three independent biological replicates. Statistical significance was assessed using two-way ANOVA, where indicated (B,D).

There was a significant decrease in the number of plaques formed by both ICI 56,780 and WR 243246-treated bradyzoites, indicating that both compounds are effective against the viability of in vitro-derived bradyzoites (Figure 4D).

### Activity of ICI 56,780 and WR 243246 against In Vivo-Derived Bradyzoites

We also tested the compounds against in vivo-derived bradyzoites, given the known differences between in vitro and in vivo derived tissue cysts.^39^ Using a previously established ex vivo protocol adapted from Radke et al.,^40^ we assessed the effects of the compounds on the viability of bradyzoites isolated from chronically infected mice. Tissue cysts were isolated from the brains of infected mice by homogenization followed by acid/pepsin treatment to release bradyzoites. The recovered bradyzoites were then used in two assays: continuous compound treatment to assess inhibition of ME49-RFP tachyzoite growth and short (4 h) exposure to evaluate effects on bradyzoite viability (Figure 5A).

**Figure 5:**
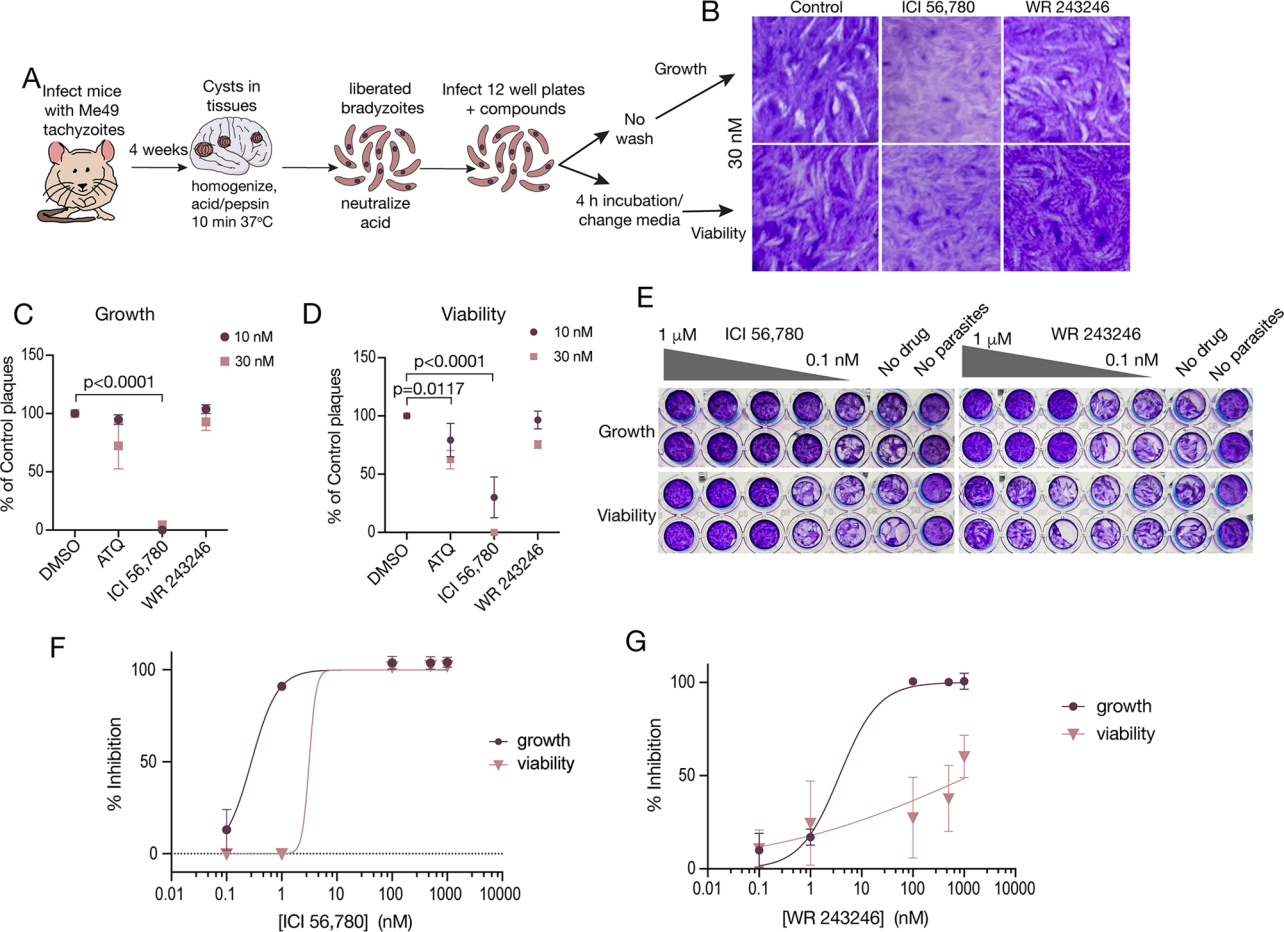
ICI 56,780 and WR 243246 inhibition of in vivo-derived bradyzoites. (A). Schematic of the protocol used for the ex vivo assay. Mice are infected with ME49 tachyzoites, and 4 weeks postinfection, brains are collected, homogenized, and cysts enumerated. Cysts are treated with acid/pepsin to liberate bradyzoites, which are used for two different assays: a growth assay in which parasites are continuously exposed to the compounds for 14 days, and a viability assay, in which bradyzoites are treated with compounds for 4 h, followed by washing and incubation in compound-free DMEM. (B). Representative images of plaques formed following the viability and growth assays. (C)**.** Average number of plaques formed in the growth assay, normalized to the DMSO-treated bradyzoites controls, calculated from 3 biological replicates. (D). Average number of plaques formed in the viability assay, normalized to the DMSO-treated bradyzoites controls, calculated from 3 biological replicates. (E). Representative plaques formed in the 96-well plate ex vivo assays for both growth and viability, showing dose–response for ICI 56,780 and WR 243246. (F). Dose response curves for ICI 56,780 in growth and viability assays from 2 independent biological replicates. (G). Dose response curves for WR 243246 in growth and viability assays from 2 independent biological replicates. EC_50_ values were determined by nonlinear regression analysis using GraphPad Prism. Statistical significance was assessed using two-way ANOVA, where indicated.

We found that only ICI 56,780 was effective at 30 nM in inhibiting both growth and viability of in vivo-derived bradyzoites (Figure 5B–D). Inhibition of bradyzoite viability provides a more direct and stage-specific measure of chronic-stage activity than inhibition of overall growth, which may primarily reflect effects on reactivated tachyzoites. We next developed a 96-well plate assay to quantify the inhibition and determine EC_50_ values. ICI 56,780 showed exceptional potency, inhibiting bradyzoite viability with an EC_50_ of 3.92 nM, higher than the EC_50_ measured for growth (0.32 nM) but still well within the low nanomolar range, indicating strong efficacy against both stages (Figure 5E, F, and Table 1). The growth EC_50_ against ME49 tachyzoites (0.32 nM) closely matches the EC_50_ against RH tachyzoites (0.34 nM), indicating that ICI 56,780 is highly effective against tachyzoites of both strains. This high potency, together with its low-nanomolar activity against bradyzoites, highlights its broad efficacy across parasite stages.

WR 243246 also inhibited ME49 tachyzoite growth effectively (EC_50_ = 3.37 nM), but its activity against bradyzoite viability was markedly reduced (EC_50_ = 1271 nM), although still measurable (Figure 5E, G, and Table 1). Thus, while WR 243246 remains active, its reduced potency toward bradyzoites contrasts with the broad, stage-spanning efficacy observed with ICI 56,780.

Altogether, these findings demonstrate that ICI 56,780 is highly effective against both in vitro and in vivo-derived bradyzoites. In contrast, WR 243246 is less effective against in vivo-derived bradyzoites despite its in vitro potency. These results underscore the importance of testing ex vivo bradyzoites to validate compound efficacy before proceeding to in vivo studies of chronic infection.

### ICI 56,780 Reduces Brain Cyst Burden in Chronically Infected Mice

Since ICI 56,780 showed strong activity against bradyzoite viability, we next evaluated its efficacy in an in vivo model of chronic *T. gondii* infection using CBA/j mice, following a previously established protocol,^16^ adapted from Doggett et al.^17^ We infected male CBA/j mice with 1,000 ME49-RFP tachyzoites i.p. and allowed the infection to progress to the chronic stage over 4 weeks. After confirming the presence of tissue cysts in two randomly selected mice, we started daily treatment for 16 days. Following a 2-week recovery period, brains were collected to enumerate tissue cysts (Figure 6A). Mice were treated with ICI 56,780 at 1 and 5 mg/kg/day, and with ATQ at 5 mg/kg/day included as a positive control. Representative images of tissue cysts stained with DBA and BAG1 are shown in Figure 6B.

**Figure 6:**
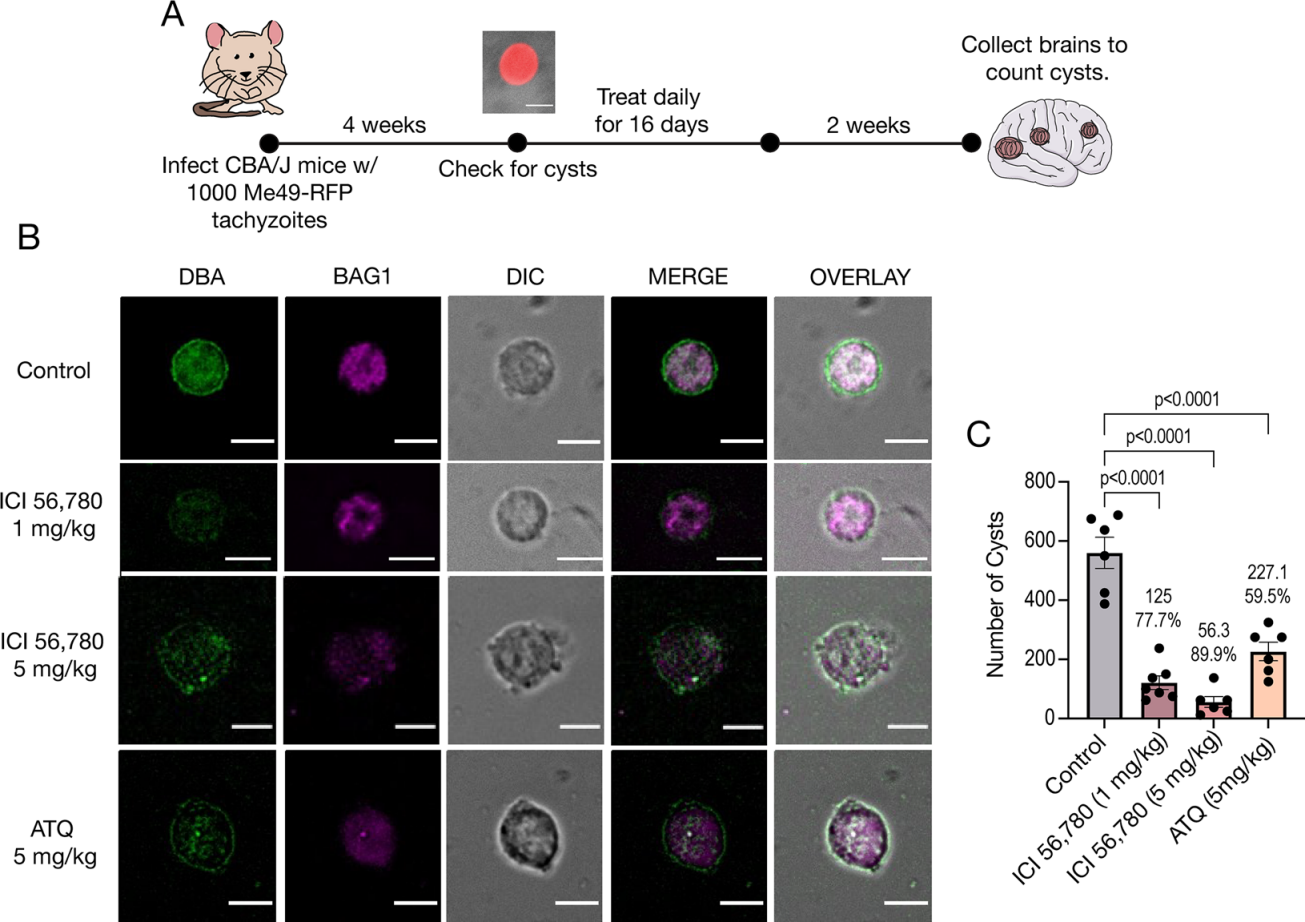
ICI 56,780 reduces brain cyst burden in a mouse model of chronic toxoplasmosis. (A). Schematic showing the protocol used for the in vivo chronic infection. CBA/j mice were infected with 1,000 Me49-RFP tachyzoites i.p. Four weeks postinfection, 2 mice were euthanized to collect their brains to confirm the presence of tissue cysts, and the remaining mice were treated daily by i.p injection for 16 days with ICI 56,780 dissolved in Kolliphor HS-15 at doses of 1 mg/kg/day and 5 mg/kg/day. ATQ was included as a positive control at a dose of 5 mg/kg/day. (B). Representative images of tissue cysts isolated from the brains of chronically infected mice treated with the indicated compounds. Cysts were stained with DBA to label the cyst wall and with anti-BAG1 antibodies to label bradyzoites. Scale bar = 5 μm. (C). Quantification of the number of cysts obtained from the brains of chronically infected and treated mice. Data represent the mean from two independent biological replicates. Each dot represents a mouse, and each group had 6 mice except the ICI 56,780 1 mg/kg group, which had a total of 7 mice. Statistical significance was assessed using two-way ANOVA, where indicated.

ICI 56,780 significantly reduced brain cyst burden by 77.7% at 1 mg/kg and 89.9% at 5 mg/kg, substantially exceeding the reduction achieved with the ATQ control (59.5%), which is consistent with the known limited efficacy of ATQ against in vivo cysts.^16^ We validated these findings with another mouse strain, Swiss Webster, which is known to be more resistant to *T. gondii* infection and, as a consequence, produces fewer tissue cysts (Figure S2A). We found that ICI 56,780 reduced cyst burden by 86.1% at 1 mg/kg and 94.4% at 5 mg/kg compared to 80.1% for ATQ (Figure S2B). All mice tested positive for the presence of ME49-RFP specific antibodies in their serum, confirming infection (Figure S2C–E).

Altogether, these results demonstrate that ICI 56,780 is highly effective at reducing cyst burden in chronically infected mice, highlighting its potential as a standalone therapeutic candidate or as a lead scaffold for developing improved treatments for chronic toxoplasmosis.

## Discussion

In this study, we investigated the antiparasitic potential of two legacy 4­(1*H*)-quinolones, ICI 56,780 and WR 243246, against *T. gondii*. We focused on evaluating their effects on mitochondrial function and assessing their efficacy against both the acute and chronic stages of infection. Both compounds demonstrated potent inhibition of tachyzoite proliferation, including activity against ATQ-resistant strains, and they disrupted mitochondrial function by inhibiting electron transport and depolarizing the mitochondrial membrane potential. Notably, ICI 56,780 exhibited subnanomolar efficacy across multiple assays, including inhibition of mitochondrial activity and reduction of bradyzoite viability in both in vitro and in vivo-derived parasites. In contrast, while WR 243246 was active against in vitro tachyzoites and bradyzoites, its effectiveness was markedly reduced against in vivo-derived bradyzoites. This observation is consistent with the physicochemical profiling of ICI 56,780 and WR 243246, which showed that ICI 56,780 has better solubility and stability. These improved physicochemical properties likely contribute to ICI 56,780s increased bioavailability compared to WR 243246. Impressively, in vivo, ICI 56,780 provided full protection in a lethal acute infection model at 10 mg/kg/day and significantly reduced brain cyst burden in chronically infected mice at low doses (1 and 5 mg/kg/day), outperforming the clinically approved ATQ. Together, these findings establish ICI 56,780 as a promising lead compound for treating both acute and chronic toxoplasmosis and highlight the importance of evaluating drug efficacy in physiologically relevant bradyzoite models. These results also support further medicinal chemistry optimization of both ICI 56,780 and WR 243246.

Mitochondrial function remains a critical vulnerability in *T. gondii* during both the tachyzoite and bradyzoite stages, making it an attractive target for therapeutic intervention against chronic infection.^4,14,17^ To assess whether these compounds target mitochondrial function via a mechanism distinct from ATQ, which inhibits the Q_o_ site of the *bc*_1_ complex, we utilized ATQ^R^ *T. gondii* strains harboring mutations in the mitochondrial cytochrome *b* gene.^7^ These strains were generated by McFadden et al. and represent a valuable tool for characterizing the mitochondrial mechanism of action of ICI 56,780 and WR 243246. The emergence of *P. falciparum* resistance to ATQ has been well documented and is often linked to point mutations in the cytochrome *b* gene, which impairs drug binding and rapidly renders treatment ineffective.^41^ The emergence of ATQ resistance is not considered a major clinical concern in *T. gondii*, largely because the parasite is not transmitted between humans; therefore, drug-resistant strains that arise during treatment are unlikely to spread widely in the population.^42^ However, between 2013 and 2017, six sulfadiazine-resistant strains were identified in Brazil, isolated either from patients with toxoplasmosis or from livestock intended for human consumption.^43^ Therefore, analyzing resistant strains is important, not only for investigating the mechanism of action of mitochondrial inhibitors but also for monitoring the potential emergence of clinically significant drug resistance. We attempted to generate ICI 56,780-resistant cell lines in three independent ENU mutagenesis experiments but were unsuccessful, as parasites failed to survive continued drug pressure after several passages.

Both ICI 56,780 and WR 243246 show comparable activity in ATQ^R^ and wild-type strains, suggesting that these quinolones inhibit mitochondrial function by engaging a site in the cytochrome *bc*_1_ complex distinct from the ATQ binding site. It has been shown that maintenance of mitochondrial membrane potential and ETC activity persists in bradyzoites and supports their long-term survival within tissue cysts.^14^ Our findings that both ICI 56,780 and WR 243246 impair mitochondrial membrane potential and inhibit ETC activity further support this therapeutic approach. These results support the mitochondrion as a druggable target during chronic infection and highlight the potential of mitochondrial inhibitors to overcome the limitations of current therapies, which fail to eliminate persistent tissue cysts. While our experiments on mitochondrial inhibition focused on tachyzoites, additional work will be required to measure mitochondrial inhibition in bradyzoites, as it is difficult to obtain sufficient numbers of parasites to perform these analyses. It is also possible that quinolones affect other enzymatic functions or metabolic processes, as has been reported in viruses, bacteria, and cancer cells.^19,44^

Building upon the established efficacy of mitochondrial inhibitors against *T. gondii*, our study highlights the advantages of 4­(1*H*)-quinolones, particularly ICI 56,780, relative to previously characterized ELQs such as ELQ-271 and ELQ-316. ELQs have shown potent in vitro activity and substantial reductions in brain cyst burden at doses of 5–25 mg/kg in murine models.^17^ ICI 56,780 achieves comparable or greater efficacy at lower doses, reducing cyst burden by 77.7% at 1 mg/kg and up to 89.9% at 5 mg/kg. These findings underscore its enhanced potency and strong potential for improved therapeutic performance. This improved in vivo performance of ICI 56,780 may be due to its distinct chemical structure compared to ELQs, though it remains unclear whether and how these structural differences influence pharmacokinetic properties that determine bioavailability and tissue penetration. Moreover, potentially distinct binding interactions of the 4­(1*H*)-quinolones ICI 56,780 and WR 243246 within the cytochrome *bc*_1_ complex may provide advantages in overcoming resistance mechanisms associated with Q_i_ inhibitors like the ELQs.^17,45^ The ability of ICI 56,780 to maintain efficacy against ATQ-resistant strains further supports its potential as a robust chemotherapeutic agent.

## Conclusions

In summary, the 4­(1*H*)-quinolone scaffolds ICI 56,780 and WR 243246 represent promising alternatives to existing mitochondrial inhibitors, combining potent anti-*T. gondii* activity with substantial medicinal chemistry optimization potential to improve physicochemical and pharmacokinetic properties. These findings support further exploration of both scaffolds for the treatment of acute and chronic toxoplasmosis.

ICI 56,780 exhibits exceptional potency across parasite stages, retains activity against ATQ-resistant strains, and achieves marked reductions in brain cyst burden at doses lower than those required for ELQ-series compounds. Its broad activity profile, spanning inhibition of electron transport, disruption of mitochondrial membrane potential, tachyzoite killing, and direct activity against in vivo-derived bradyzoites, highlights advantages over current clinical agents such as pyrimethamine-sulfadiazine, which has little activity against tissue cysts, or ATQ, which reduces but does not eliminate encysted parasites. The ability of ICI 56,780 to maintain efficacy in the presence of *bc*_1_ mutations associated with ATQ resistance further highlights its potential as a next-generation mitochondrial inhibitor that may overcome limitations of present-day treatment regimens.

Together, these findings identify ICI 56,780 as a strong lead scaffold for further pharmacokinetic optimization and structure-guided medicinal chemistry refinement. Future work integrating mechanism-of-action studies and combination therapy approaches will be essential for defining the translational potential of this compound class. More broadly, this study reinforces the value of targeting parasite mitochondrial function, an essential activity maintained in bradyzoites, as a viable strategy for achieving more effective, possibly sterilizing, therapies for chronic toxoplasmosis.

## Materials and Methods

### Ethics Statement

All animal care and therapy studies were carried out in accordance with the NIH guidelines. The animal use protocol was reviewed and approved by the Institutional Animal Care and Use Committee (IACUC) of the University of Georgia. AUP# A2021 03-005-Y3-A4 and A2024 03-021Y2-A2.

### Cultures

*T. gondii* RH strain (type I strain) was cultured using hTERT (human telomerase reverse transcriptase) cells with 1% bovine calf serum (BCS) and purified as described.^46^ *T. gondii* ME49 (type II strain) were cultured using HFF (human foreskin fibroblasts) cells with 1% BCS. The ME49 parental strain was transfected with a Td-Tomato plasmid to create the ME49-RFP strain. Host cells were grown in Dulbecco’s modified Eagle medium supplemented with 10% BCS (hTERT) or 10% FBS (HFF). Cell cultures were maintained at 37 °C with 5% CO_2_.

### Physicochemical Property Analysis

Physicochemical property experiments were performed by TCG Lifesciences (Kolkata, India). Determination of physicochemical properties and data analysis were performed as previously reported.^47^

### Synthesis of ICI 56,780 and WR 243246

#### General

Unless otherwise noted, all reagents and solvents were purchased from commercial sources and used without further purification. Tetrahydrofuran (THF) was distilled from benzophenone and sodium metal under an argon atmosphere immediately before use. Column chromatography was carried out using Sorbtec silica gel 60 Å (particle size 40–63 μm), and analytical thin-layer chromatography was performed on 0.25 mm silica gel 60 F254 precoated plates from EMD Millipore. Microwave reactions were performed in an Anton Paar Monowave 400. The identity of all title compounds was verified via proton nuclear magnetic resonance (^1^H NMR) spectroscopy and liquid chromatography with low-resolution mass spectrometry detection (LRMS). ^1^H NMR spectra were recorded at ambient temperature on a Bruker 500 or 700 MHz spectrometer. All ^1^H NMR experiments are reported in δ units, parts per million (ppm) downfield of trimethyl silane (TMS) and were measured relative to the residual proton signals of chloroform (δ 7.26) and dimethylsulfoxide (δ 2.50). Data for ^1^H NMR are reported as follows: chemical shift (δ ppm), multiplicity (bs = broad singlet, s = singlet, d = doublet, dd = doublet of doublets, dt = doublet of triplets, ddt = doublet of doublet of triplets, t = triplet, tt = triplet of triplets, q = quartet, hept = heptet, m = multiplet), coupling constant (Hz), and integration. NMR data was analyzed by using MestReNova Software version 12.0.3–21,384. Low resolution mass spectra (LRMS) were acquired on an Agilent 1260 Infinity HPLC with a thermostated well-plate autosampler and diode array detector hyphenated to an Agilent G6125B single quadrupole mass spectrometer with electrospray ionization. At a flow rate of 0.6 mL/min, samples were analyzed on an Agilent ZORBAX RRHT StableBond-C18 column (1.8 μm, 2.1 × 50 mm, part no: 827700-902) with the following HPLC method: 0 min 90/10 A/B; 3.5 min 10/90 A/B; 5.5 min 10/90 A/B; and 6.0 min 90/10 A/B, where A consisted of water (+0.1% formic acid) and B consisted of acetonitrile (+0.1% formic acid). All final compounds were >95% pure by HPLC analysis.

#### Synthesis of ICI 56,780

ICI 56,780 was synthesized as previously reported.^29^ The crude material was purified via recrystallization in dichloromethane to afford a white solid. ^1^H NMR (500 MHz, DMSO-*d*_6_): δ 12.11 (s, 1H), 8.49 (s, 1H), 7.88 (s, 1H), 7.31 (dd, *J* = 7.8 Hz, 2H), 7.06 (s, 1H), 7.02–6.94 (m, 3H), 4.41 (s, 4H), 3.73 (s, 3H), 2.62 (t, *J* = 7.6 Hz, 2H), 1.53 (p, *J* = 7.4 Hz, 2H), 1.29–1.22 (m, 2H), 0.84 (t, *J* = 7.3 Hz, 3H). LRMS-ESI (*m*/*z*): [M + H]^+^ 396.2; retention time: 3.92 min. TLC (5% methanol in dichloromethane) *R*_f_ 0.41 (UV, 254 nm, 280 nm).

#### Synthesis WR 243246

WR 243246 was synthesized as previously reported.^28^ The crude material was purified via recrystallization in dichloromethane to afford a yellow solid. ^1^H NMR (500 MHz, Chloroform-*d*): δ 9.77 (s, 1H), 8.49 (d, *J* = 2.1 Hz, 1H), 8.14–8.04 (m, 2H), 7.50 (d, *J* = 2.1 Hz, 1H), 7.35 (dd, *J* = 8.4, 2.1 Hz, 1H), 7.25 (s, 1H), 4.20–4.09 (m, 1H), 3.79–3.63 (m, 2H), 3.27–3.19 (m, 1H), 3.17–3.07 (m, 1H). LRMS-ESI (*m*/*z*): [M + H]^+^ 391.6; retention time 3.27 min. TLC (5% methanol in dichloromethane) *R*_f_ 0.38 (UV, 254 nm, 280 nm).

### In vitro Dose Response Curves

Experiments with *T. gondii* tachyzoites were carried out with parasites expressing a td-Tomato red fluorescent protein (RFP).^48,49^ Parasites were purified by passing them through a 25-gauge needle, followed by filtration through a 5 μm filter. Human fibroblasts were cultured in 96-well black plates for 48 (hTERT) or 120 (HFF) hours before the addition of 4,000 fluorescent tachyzoites/well. Compounds were tested at five different concentrations to generate dose–response curves for ICI 56,780 and WR 243246, while ATQ was tested at ten different concentrations. Two technical replicates per concentration were used, and the experiment was repeated in three independent biological replicates. Fluorescence values were measured for up to 7 days, and both excitation (544 nm) and emission (590 nm) were read from the bottom of the plates in a BioTek Synergy H1 Hybrid plate reader.^16^ For the dose–response curves of the ATQ^R^ strains, which are nonfluorescent, parasites were allowed to grow for 7 days before staining the plates with crystal violet and reading the absorbance of the wells at 590 nm using a BioTek Synergy H1 plate reader. The EC_50_s were calculated using GraphPad Prism software version 10.

### Cytochrome *c* Reduction Assay

The assay was carried out using a previously published protocol.^50^ Five μg of protein from a *T. gondii* mitochondrial-enriched fraction (P2) was used for each technical replicate (3 in total). After the addition of reaction buffer (0.3 mM succinate, 0.3 mM KCN, 10 mM HEPES, pH 7.5), with succinate as the substrate and each compound at 100 nM, the reaction was initiated by the addition of cytochrome *c* (0.1 mM final concentration). The absorbance of reduced cytochrome *c* was measured at 550 nm using a BioTek Synergy H1 plate reader for 10 min with shaking at 30 °C. The activity of each fraction was then normalized to protein concentration as determined by the BCA assay.

### Mitochondrial Membrane Potential

The mitochondrial membrane potential was measured by the safranine O method according to published protocols.^34,35^ Freshly lysed parasites were collected and filtered through an 8 μm filter to remove host cell debris. The parasites were washed twice with BAG (116 mM NaCl, 5.4 mM KCl, 0.8 mM MgSO_4_·7H_2_O, 50 mM HEPES, 5.5 mM Glucose, pH 7.3) and resuspended at 1 × 10^9^ parasites per ml. An aliquot of 50 μL of the parasite suspension (5 × 10^7^ cells) was added to a cuvette containing Safranine O (2.5 μM) and succinate (1 mM) in 2 mL of reaction buffer (125 mM sucrose, 65 mM KCl, 10 mM HEPES-KOH pH 7.2, 1.0 mM MgCl_2_, and 2.5 mM K_3_PO_4_ pH 7.2). The cuvette was placed in a Hitachi F-7000 fluorescence spectrophotometer, and digitonin (10 μM) was added to selectively permeabilize the plasma membrane. After equilibration, ADP (10 μM final) was added to stimulate oxidative phosphorylation, followed by the compounds at 2.5 μM (ATQ) and 1 μM (ICI 56,780 and WR 243246). The change in mitochondrial membrane potential was quantified as the difference between the maximum fluorescence (after compound addition) and the minimum fluorescence (following ADP stabilization and before compound addition). We used similar conditions to measure the mitochondrial membrane potential of mammalian cells, with the following modifications: hTert cells were resuspended at 10^8^ cells/mL, 5 mM glutamate and 5 mM malate were used as substrates, 50 μM digitonin was used for permeabilization, and compounds were added 400 s after permeabilization. Statistical analysis was performed using two-way ANOVA in GraphPad Prism 10.

### Acute In Vivo Infection in Mice

Experiments were carried out as described previously^51^ using 100 *T. gondii* tachyzoites of the RH td-Tomato strain (RH-RFP) to infect Swiss Webster mice. Drugs were dissolved in 10% Kolliphor HS-15, and mice were inoculated intraperitoneally (i.p.) starting 6 h after infection and administered for 10 days. The survival of mice was monitored along with their weight. Mice were sacrificed if they lost over 20% of their body weight. Surviving mice were challenged on day 30 postinfection with 10,000 RH-RFP tachyzoites. Blood was collected at 28 dpi (before challenge) and at the time of euthanasia (3 weeks post challenge) to analyze the presence of *T. gondii* antibodies. To test for antibodies, we performed Western blots with RH-RFP lysates and probed the membrane with serum collected from the surviving mice. Protein extraction and Western blot conditions were done as previously.^15^ After blocking with 5% milk in PBS-T (0.1% tween 20 in PBS), the membranes were washed 3x for 5 min with PBS-T and attached to the BioRad multiscreen tool. The RH-RFP lysate was run across the entire gel to allow the testing of different sera (antibodies) on the same membrane. We incubated the membrane with serum (used as the primary antibody) for 1 h in the multiscreen tool and then washed the membrane five times for 3 min each while still in the multiscreen, followed by one additional wash after removal. Then we used IRDye 800 CW goat anti-mouse IgG secondary antibody and incubated for 1 h before washing three times with PBS-T and imaging the blot on a LI-COR Odyssey CLx at 800 nm.

### In Vitro Bradyzoite Differentiation and Immunofluorescence Assay

Type II ME49 tachyzoites were differentiated using compound 1 (4-[2-(4-fluorophenyl)-5-(1-methylpiperidine-4-yl)-1H-pyrrol-3-yl]­pyridine) as previously described.^52^ Confluent HFF monolayers on glass coverslips (preincubated for 2 h with 3 μM compound 1, as described^37^) were infected with 2 × 10^5^ tachyzoites of the ME49 strain. The cells were allowed to grow and differentiate in a 37 °C under ambient CO_2_ for 3 days. The cultures were then treated with compounds at 3x their EC_50_ for an additional 4 days at ambient CO_2_. After 7 total days, the cells were fixed with 3% paraformaldehyde for 10 min, permeabilized with 0.25% triton X-100 for 10 min, and blocked in 3% bovine serum albumin (BSA) for 1 h. The coverslips were then incubated with rhodamine-conjugated *D. biflorus* agglutinin (DBA; 1:2,000),^38^ rabbit anti-BAG1 antibody (1:1,000), and rat anti-SAG1 antibody (1:1,000) (both a kind gift from Vern Carruthers) for 2 h. Then the secondary anti-rat 488 and anti-rabbit 633 were used to visualize the SAG1 antibody. The cells were then mounted onto microscope slides with fluoromount and DAPI. The slides were placed in the fridge for a minimum of 24 h before imaging on a Delta Vision microscope. The vacuole sizes were measured using ImageJ software.

### In Vitro Bradyzoite Viability Assay

The protocol for in vitro viability assay was adapted from Zwicker et al.^54^ Confluent HFF monolayers (preincubated for 2 h with 3 μM compound 1) were infected with 2.5 × 10^5^ tachyzoites of the ME49 (type II) strain. After 24 h, compound 1 was removed to avoid toxicity to the host cells. Parasites were allowed to grow and differentiate at 37 °C under ambient CO_2_ for 3 days. The cysts were then incubated with drugs at 30 nM for an additional 4 days at ambient CO_2_. After 7 days of differentiation, the cysts were collected by scraping the monolayers, syringe lysing the parasites, and subsequently treating them with acid/pepsin (170 mM sodium chloride, 60 mM hydrochloric acid, and 0.1 mg/mL pepsin) for 30 min in a 37 °C water bath. The parasite suspension was neutralized with 94 mM NA_2_CO_3_ and enumerated. 10,000 bradyzoites were plated per well in a 6-well plate and allowed to grow undisturbed in a 37 °C incubator with 5% CO_2_ for 16 days. At this time, cultures were fixed with 100% ethanol and stained with 2.5× crystal violet. Plaques were quantified using a light microscope using the 4× objective and were enumerated and normalized as a percentage of the plaques formed by the DMSO control.

### Isolation and Ex Vivo Assay of In Vivo-Derived Bradyzoites

Brains of mice chronically infected with ME49 tachyzoites i.p. were collected 28 dpi, homogenized with 7 strokes in a glass homogenizer, and then enumerated on a Delta Vision microscope (protocol adapted from^40^). For 12 well plate assays, 25 cysts per plate were placed in a tube for acid/pepsin (170 mM sodium chloride, 60 mM hydrochloric acid, and 0.1 mg/mL pepsin) treatment for 10 min in a 37 °C water bath. The cyst suspension was then neutralized with 94 mM sodium carbonate (NA_2_CO_3_). The liberated bradyzoites were then equally distributed into the wells of a 12-well plate. For the growth assay, the cells were grown in the presence of compounds or DMSO for 12 and 14 days, undisturbed. For the viability assays, the cells were treated with compounds or DMSO for 4 h, washed with 1× PBS, and then grown for 14 days in compound-free media. After 14 days, the cells were fixed with 100% ethanol and stained with 2.5× crystal violet. We also adapted this assay to a 96-well plate format to generate dose–response curves. In this format, 500 cysts were seeded per plate, and both growth and viability assays were performed as described above. Plaques from the 12-well plates were quantified on a light microscope using the 4× objective, and the size of the plaques was measured using Fiji (ImageJ) software. For the 96-well plate assays, we measured the absorbance at 590 using a BioTek Synergy H1 plate reader. Dose-response curves were calculated using GraphPad Prism software version 10.

### Chronic *Toxoplasma* Infection in Mice

CBA/j mice (Jackson Laboratory) were infected i.p. with a 200 μL suspension of 1,000 tachyzoites of the ME49-RFP strain *T. gondii* (type II genotype).^53^ About 15% of the mice succumbed to the acute infection before 28 days post-infection. At 4 weeks post-infection, the brains of 2 mice were collected to check for the presence of cysts and to confirm that the mice had entered the chronic stage. At this time, the remaining mice were injected i.p. daily for 16 consecutive days with 200 μL of solvent (10% kolliphor HS-15) or of compounds dissolved in 10% HS-15. ATQ was administered at 5 mg/kg, and ICI 56,780 was administered at 1 mg/kg or 5 mg/kg. Mice were euthanized humanely 2 weeks after the final treatment. The mouse brains were collected in 1 mL sterile PBS, minced with scissors, and homogenized using a glass homogenizer. 420 μL samples (80 μL total or 8% of the sample) of each brain homogenate were placed on a glass microscope slide. Cysts were enumerated in a Delta vision fluorescence microscope.

### Statistical Analysis

Experimental data are expressed as the mean values ±standard error of the mean (SEM) from at least 3 independent biological replicates unless indicated otherwise. Statistical analyses were performed using two-way Anova statistical analysis using GraphPad PRISM version 10. We used Kaplan-Meier survival analysis for in vivo virulence significance. A *P*-value of <0.05 was considered statistically significant.

## Supplementary Material



## Notes

Cytotoxicity data in HFF and BV-2 cells; additional data from acute in vivo infection studies, including serum antibody measurements and body weight analyses; additional chronic in vivo infection data from Swiss Webster mice treated with ICI 56,780 (PDF)

## References

[1] Hill D., Dubey J. P. (2002). Toxoplasma
gondii: transmission, diagnosis and prevention. Clin. Microbiol. Infect..

[2] Tenter A. M., Heckeroth A. R., Weiss L. M. (2000). Toxoplasma gondii: from animals to
humans. Int. J. Parasitol..

[3] Schoondermark-van
de Ven E., Vree T., Melchers W., Camps W., Galama J. (1995). In vitro effects of sulfadiazine and its metabolites
alone and in combination with pyrimethamine on Toxoplasma gondii. Antimicrob. Agents Chemother..

[4] Alday P. H., Bruzual I., Nilsen A., Pou S., Winter R., Ben Mamoun C., Riscoe M. K., Doggett J. S. (2017). Genetic Evidence
for Cytochrome b Qi Site Inhibition by 4­(1H)-Quinolone-3-Diarylethers
and Antimycin in Toxoplasma gondii. Antimicrob.
Agents Chemother..

[5] Dunay I. R., Gajurel K., Dhakal R., Liesenfeld O., Montoya J. G. (2018). Treatment of Toxoplasmosis: Historical Perspective,
Animal Models, and Current Clinical Practice. Clin. Microbiol. Rev..

[6] Fry M., Pudney M. (1992). Site of action of the antimalarial hydroxynaphthoquinone,
2-[trans-4-(4’-chlorophenyl) cyclohexyl]-3-hydroxy-1,4-naphthoquinone
(566C80). Biochem. Pharmacol..

[7] McFadden D. C., Tomavo S., Berry E. A., Boothroyd J. C. (2000). Characterization
of cytochrome b from Toxoplasma gondii and Q­(o) domain mutations as
a mechanism of atovaquone-resistance. Mol. Biochem.
Parasitol..

[8] Ferguson D. J., Huskinson-Mark J., Araujo F. G., Remington J. S. (1994). An ultrastructural
study of the effect of treatment with atovaquone in brains of mice
chronically infected with the ME49 strain of Toxoplasma gondii. Int. J. Exp. Pathol..

[9] Tu V., Yakubu R., Weiss L. M. (2018). Observations on bradyzoite biology. Microbes Infect..

[10] Weiss L. M., Kim K. (2000). The development and biology of bradyzoites
of Toxoplasma gondii. Front Biosci..

[11] Sinai A. P., Watts E. A., Dhara A., Murphy R. D., Gentry M. S., Patwardhan A. (2016). Reexamining Chronic Toxoplasma gondii Infection: Surprising
Activity for a ″Dormant″ Parasite. Curr. Clin Microbiol Rep.

[12] Araujo F. G., Huskinson-Mark J., Gutteridge W. E., Remington J. S. (1992). In vitro
and in vivo activities of the hydroxynaphthoquinone 566C80 against
the cyst form of Toxoplasma gondii. Antimicrob.
Agents Chemother..

[13] Watts E., Zhao Y., Dhara A., Eller B., Patwardhan A., Sinai A. P. (2015). Novel Approaches Reveal that Toxoplasma gondii Bradyzoites
within Tissue Cysts Are Dynamic and Replicating Entities In Vivo. mBio.

[14] Place B. C., Troublefield C. A., Murphy R. D., Sinai A. P., Patwardhan A. R. (2023). Machine
learning based classification of mitochondrial morphologies from fluorescence
microscopy images of Toxoplasma gondii cysts. PLoS One.

[15] Sleda M. A., Li Z. H., Behera R., Baierna B., Li C., Jumpathong J., Malwal S. R., Kawamukai M., Oldfield E., Moreno S. N. J. (2022). The Heptaprenyl Diphosphate Synthase
(Coq1) Is the Target of a Lipophilic Bisphosphonate That Protects
Mice against Toxoplasma gondii Infection. mBio.

[16] Sleda M. A., Pitafi Z. F., Song W., Oldfield E., Moreno S. N. J. (2024). Lipophilic
bisphosphonates reduced cyst burden and ameliorated hyperactivity
of mice chronically infected with Toxoplasma gondii. mBio.

[17] Doggett J. S., Nilsen A., Forquer I., Wegmann K. W., Jones-Brando L., Yolken R. H., Bordon C., Charman S. A., Katneni K., Schultz T., Burrows J. N., Hinrichs D. J., Meunier B., Carruthers V. B., Riscoe M. K. (2012). Endochin-like quinolones are highly
efficacious against acute and latent experimental toxoplasmosis. Proc. Natl. Acad. Sci. U. S. A..

[18] Doggett J. S., Schultz T., Miller A. J., Bruzual I., Pou S., Winter R., Dodean R., Zakharov L. N., Nilsen A., Riscoe M. K., Carruthers V. B. (2020). Orally Bioavailable Endochin-Like
Quinolone Carbonate Ester Prodrug Reduces Toxoplasma gondii Brain
Cysts. Antimicrob. Agents Chemother..

[19] Dalhoff A. (2015). Antiviral,
antifungal, and antiparasitic activities of fluoroquinolones optimized
for treatment of bacterial infections: a puzzling paradox or a logical
consequence of their mode of action?. Eur. J.
Clin. Microbiol. Infect. Dis..

[20] Sharma V., Das R., Kumar Mehta D., Gupta S., Venugopala K. N., Mailavaram R., Nair A. B., Shakya A. K., Kishore
Deb P. (2022). Recent insight into the biological activities and SAR of quinolone
derivatives as multifunctional scaffold. Bioorg.
Med. Chem..

[21] Sharma V., Das R., Mehta D. K., Sharma D., Sahu R. K. (2022). Exploring Quinolone
Scaffold: Unravelling the Chemistry of Anticancer Drug Design. Mini Rev. Med. Chem..

[22] Kohanski M. A., Dwyer D. J., Hayete B., Lawrence C. A., Collins J. J. (2007). A common
mechanism of cellular death induced by bactericidal antibiotics. Cell.

[23] Uivarosi V. (2013). Metal complexes
of quinolone antibiotics and their applications: an update. Molecules.

[24] Maignan J. R., Lichorowic C. L., Giarrusso J., Blake L. D., Casandra D., Mutka T. S., LaCrue A. N., Burrows J. N., Willis P. A., Kyle D. E., Manetsch R. (2016). ICI 56,780
Optimization: Structure-Activity
Relationship Studies of 7-(2-Phenoxyethoxy)-4­(1H)-quinolones with
Antimalarial Activity. J. Med. Chem..

[25] Cross R. M., Monastyrskyi A., Mutka T. S., Burrows J. N., Kyle D. E., Manetsch R. (2010). Endochin optimization:
structure-activity and structure-property
relationship studies of 3-substituted 2-methyl-4­(1H)-quinolones with
antimalarial activity. J. Med. Chem..

[26] Nilsen A., LaCrue A. N., White K. L., Forquer I. P., Cross R. M., Marfurt J., Mather M. W., Delves M. J., Shackleford D. M., Saenz F. E., Morrisey J. M., Steuten J., Mutka T., Li Y., Wirjanata G., Ryan E., Duffy S., Kelly J. X., Sebayang B. F., Zeeman A. M., Noviyanti R., Sinden R. E., Kocken C. H. M., Price R. N., Avery V. M., Angulo-Barturen I., Jimenez-Diaz M. B., Ferrer S., Herreros E., Sanz L. M., Gamo F. J., Bathurst I., Burrows J. N., Siegl P., Guy R. K., Winter R. W., Vaidya A. B., Charman S. A., Kyle D. E., Manetsch R., Riscoe M. K. (2013). Quinolone-3-diarylethers:
a new class of antimalarial drug. Sci. Transl.
Med..

[27] Cross R. M., Flanigan D. L., Monastyrskyi A., LaCrue A. N., Saenz F. E., Maignan J. R., Mutka T. S., White K. L., Shackleford D. M., Bathurst I., Fronczek F. R., Wojtas L., Guida W. C., Charman S. A., Burrows J. N., Kyle D. E., Manetsch R. (2014). Orally bioavailable
6-chloro-7-methoxy-4­(1H)-quinolones efficacious against multiple stages
of Plasmodium. J. Med. Chem..

[28] Cross R. M., Maignan J. R., Mutka T. S., Luong L., Sargent J., Kyle D. E., Manetsch R. (2011). Optimization of 1,2,3,4-tetrahydroacridin-9­(10H)-ones
as antimalarials utilizing structure-activity and structure-property
relationships. J. Med. Chem..

[29] Cross R. M., Namelikonda N. K., Mutka T. S., Luong L., Kyle D. E., Manetsch R. (2011). Synthesis, antimalarial activity, and structure-activity
relationship of 7-(2-phenoxyethoxy)-4­(1H)-quinolones. J. Med. Chem..

[30] Neelarapu R., Maignan J. R., Lichorowic C. L., Monastyrskyi A., Mutka T. S., LaCrue A. N., Blake L. D., Casandra D., Mashkouri S., Burrows J. N., Willis P. A., Kyle D. E., Manetsch R. (2018). Design and Synthesis of Orally Bioavailable Piperazine
Substituted 4­(1H)-Quinolones with Potent Antimalarial Activity: Structure-Activity
and Structure-Property Relationship Studies. J. Med. Chem..

[31] Maclean A. E., Hayward J. A., Huet D., van Dooren G. G., Sheiner L. (2022). The mystery of massive mitochondrial complexes: the
apicomplexan respiratory chain. Trends Parasitol..

[ref31b] Usey M. M., Huet D. (2022). Parasite powerhouse:
A review of
the Toxoplasma gondii mitochondrion. J. Eukaryot
Microbiol.

[32] Acharjee R., Talaam K. K., Hartuti E. D., Matsuo Y., Sakura T., Gloria B. M., Hidano S., Kido Y., Mori M., Shiomi K., Sekijima M., Nozaki T., Umeda K., Nishikawa Y., Hamano S., Kita K., Inaoka D. K. (2021). Biochemical
Studies of Mitochondrial Malate: Quinone Oxidoreductase from Toxoplasma
gondii. Int. J. Mol. Sci..

[33] Jeffers V., Kamau E. T., Srinivasan A. R., Harper J., Sankaran P., Post S. E., Varberg J. M., Sullivan W. J., Boyle J. P. (2017). TgPRELID, a Mitochondrial Protein Linked to Multidrug
Resistance in the Parasite Toxoplasma gondii. mSphere.

[34] Vercesi A. E., Rodrigues C. O., Uyemura S. A., Zhong L., Moreno S. N. (1998). Respiration
and oxidative phosphorylation in the apicomplexan parasite Toxoplasma
gondii. J. Biol. Chem..

[35] McPhillie M. J., Zhou Y., Hickman M. R., Gordon J. A., Weber C. R., Li Q., Lee P. J., Amporndanai K., Johnson R. M., Darby H., Woods S., Li Z. H., Priestley R. S., Ristroph K. D., Biering S. B., El Bissati K., Hwang S., Hakim F. E., Dovgin S. M., Lykins J. D., Roberts L., Hargrave K., Cong H., Sinai A. P., Muench S. P., Dubey J. P., Prud’homme R. K., Lorenzi H. A., Biagini G. A., Moreno S. N., Roberts C. W., Antonyuk S. V., Fishwick C. W. G., McLeod R. (2020). Potent Tetrahydroquinolone
Eliminates Apicomplexan Parasites. Front. Cell.
Infect. Microbiol..

[36] Araujo F. G., Huskinson J., Remington J. S. (1991). Remarkable in vitro and in vivo activities
of the hydroxynaphthoquinone 566C80 against tachyzoites and tissue
cysts of Toxoplasma gondii. Antimicrob. Agents
Chemother..

[37] Radke J. R., Donald R. G., Eibs A., Jerome M. E., Behnke M. S., Liberator P., White M. W. (2006). Changes in the expression of human
cell division autoantigen-1 influence Toxoplasma gondii growth and
development. PLoS Pathog..

[38] Tobin C., Pollard A., Knoll L. (2010). Toxoplasma
gondii cyst wall formation
in activated bone marrow-derived macrophages and bradyzoite conditions. J. Vis Exp.

[39] Mayoral J., Di Cristina M., Carruthers V. B., Weiss L. M. (2020). Toxoplasma gondii:
Bradyzoite Differentiation In Vitro and In Vivo. Methods Mol. Biol..

[40] Radke J. B., Melillo B., Mittal P., Sharma M., Sharma A., Fu Y., Uddin T., Gonse A., Comer E., Schreiber S. L., Gupta A. K., Chatterjee A. K., Sibley L. D. (2022). Bicyclic azetidines
target acute and chronic stages of Toxoplasma gondii by inhibiting
parasite phenylalanyl t-RNA synthetase. Nat.
Commun..

[41] Korsinczky M., Chen N., Kotecka B., Saul A., Rieckmann K., Cheng Q. (2000). Mutations in Plasmodium
falciparum cytochrome b that are associated
with atovaquone resistance are located at a putative drug-binding
site. Antimicrob. Agents Chemother..

[42] McFadden D. C., Camps M., Boothroyd J. C. (2001). Resistance
as a tool in the study
of old and new drug targets in Toxoplasma. Drug
Resist Updat.

[43] Montazeri M., Mehrzadi S., Sharif M., Sarvi S., Tanzifi A., Aghayan S. A., Daryani A. (2018). Drug Resistance
in Toxoplasma gondii. Front. Microbiol..

[44] Zhou J., Chen Q., Ren R., Yang J., Liu B., Horton J. R., Chang C., Li C., Maksoud L., Yang Y., Rotili D., Zhang X., Blumenthal R. M., Chen T., Gao Y., Valente S., Mai A., Cheng X. (2024). Quinoline-based compounds can inhibit diverse enzymes that act on
DNA. Cell Chem. Biol..

[45] McConnell E. V., Bruzual I., Pou S., Winter R., Dodean R. A., Smilkstein M. J., Krollenbrock A., Nilsen A., Zakharov L. N., Riscoe M. K., Doggett J. S. (2018). Targeted Structure-Activity Analysis
of Endochin-like Quinolones Reveals Potent Qi and Qo Site Inhibitors
of Toxoplasma gondii and Plasmodium falciparum Cytochrome bc(1) and
Identifies ELQ-400 as a Remarkably Effective Compound against Acute
Experimental Toxoplasmosis. ACS Infect. Dis..

[46] Vella S. A., Calixto A., Asady B., Li Z. H., Moreno S. N. J. (2020). Genetic
Indicators for Calcium Signaling Studies in Toxoplasma gondii. Methods Mol. Biol..

[47] Wagner A., Trombley R., Podgurski M., Ruberto A. A., Cui M., Cooper C. A., Long W. E., Nguyen G. B., Marin A. A., Mai S. L., Lombardo F., Maher S. P., Kyle D. E., Manetsch R. (2025). Discovery and optimization
of a novel carboxamide scaffold
with selective antimalarial activity. Eur. J.
Med. Chem..

[48] van
Dooren G. G., Tomova C., Agrawal S., Humbel B. M., Striepen B. (2008). Toxoplasma gondii Tic20 is essential for apicoplast
protein import. Proc. Natl. Acad. Sci. U. S.
A..

[49] Recher M., Barboza A. P., Li Z. H., Galizzi M., Ferrer-Casal M., Szajnman S. H., Docampo R., Moreno S. N., Rodriguez J. B. (2013). Design,
synthesis and biological evaluation of sulfur-containing 1,1-bisphosphonic
acids as antiparasitic agents. Eur. J. Med.
Chem..

[50] Baierna B., Rahman T., Latimer S., Basset G. J., Moreno S. N. J. (2026). Evolutionary
remodeling of ubiquinone biosynthesis in Toxoplasma gondii reveals
an essential bi-functional monooxygenase. Nat.
Commun..

[51] Li Z. H., Ramakrishnan S., Striepen B., Moreno S. N. (2013). Toxoplasma gondii
relies on both host and parasite isoprenoids and can be rendered sensitive
to atorvastatin. PLoS Pathog..

[52] Radke J. R., Behnke M. S., Mackey A. J., Radke J. B., Roos D. S., White M. W. (2005). The transcriptome
of Toxoplasma gondii. BMC Biol..

[53] Pollard A. M., Knoll L. J., Mordue D. G. (2009). The role of specific Toxoplasma gondii
molecules in manipulation of innate immunity. Trends Parasitol..

[54] Zwicker J. D., Smith D., Guerra A. J., Hitchens J. R., Haug N., Vander Roest S., Lee P., Wen B., Sun D., Wang L., Keep R. F., Xiang J., Carruthers V. B., Larsen S. D. (2020). Discovery and Optimization of Triazine Nitrile Inhibitors
of Toxoplasma gondii Cathepsin L for the Potential Treatment of Chronic
Toxoplasmosis in the CNS. ACS Chem. Neurosci..

